# General ability and specific cognitive functions are lower in children with epilepsy after perinatal ischemic stroke

**DOI:** 10.3389/fstro.2024.1371093

**Published:** 2024-05-13

**Authors:** Ulvi Vaher, Mairi Männamaa, Rael Laugesaar, Norman Ilves, Nigul Ilves, Dagmar Loorits, Pille Kool, Pilvi Ilves

**Affiliations:** ^1^Department of Radiology, Institute of Clinical Medicine, University of Tartu, Tartu, Estonia; ^2^Children's Clinic, Tartu University Hospital, Tartu, Estonia; ^3^Department of Pediatrics, Institute of Clinical Medicine, University of Tartu, Tartu, Estonia; ^4^Radiology Clinic, Tartu University Hospital, Tartu, Estonia

**Keywords:** epilepsy, interictal epileptiform discharges, ischemic perinatal stroke, general ability, specific cognitive functions

## Abstract

**Introduction:**

Epilepsy develops in one third of children after perinatal stroke. Both epilepsy and stroke may be risk factors for impaired cognitive abilities. How the development of epilepsy is related to the cognitive profile of children with perinatal stroke is still unclear. The aim of the study was to evaluate general and specific cognitive functions in children with epilepsy and children without epilepsy after perinatal ischemic stroke.

**Methods:**

The study group consisted of 51 children with perinatal ischemic stroke confirmed by magnetic resonance imaging: 27 (53%) children with arterial ischemic stroke and 24 (47%) with periventricular venous infarction. Magnetic resonance imaging and electroencephalography were performed in all patients after the neonatal period. Epilepsy was diagnosed if the child had at least two unprovoked seizures occurring >24 h apart or one unprovoked seizure with a high recurrence risk. Cognitive assessments were performed using the Kaufman Assessment Battery for Children, Second Edition, at the age of ≥7 years. General ability (Fluid Crystallized Index, Mental Processing Index, Non-verbal Index) and specific cognitive functions (sequential processing, simultaneous processing, learning, planning, knowledge) were evaluated.

**Results:**

At the median age of 19.3 years (interquartile range 14.0–22) at the time of follow-up for epilepsy, 14 (27.5%) patients had developed epilepsy, and 37 (72.5%) patients were without epilepsy. All general cognitive ability scores were lower in children with epilepsy compared to children without epilepsy. Among specific cognitive functions, simultaneous processing, planning, and knowledge were lower in children with epilepsy compared to children without epilepsy: simultaneous processing mean [78.5, 95% CI: [69.8, 87.2], vs. 96.9, 95% CI [90, 103.9], *p* = 0.0018]; planning mean [82.5, 95% CI: [73, 92], vs. 96.2, 95% CI: [88.7, 103.6], *p* = 0.026]; knowledge median (25th, 75th percentile): 80.5 (75, 87) vs. 92 (84, 108), *p* = 0.023.

**Conclusion:**

Children with epilepsy after perinatal ischemic stroke have lower general cognitive abilities compared to children without epilepsy. The profile of the subscales indicates lower verbal abilities and executive functions in children with epilepsy. Children with post-stroke epilepsy need targeted cognitive monitoring for early aimed rehabilitation and for establishing an adapted learning environment.

## Introduction

Perinatal stroke is a focal vascular brain injury occurring between the 20th week of fetal life and the 28th postnatal day (Raju et al., 2007). According to the occlusion of the vessel type, perinatal ischemic stroke can be classified as arterial ischemic stroke (AIS) or periventricular venous infarction (PVI) (Raju et al., 2007; Kirton et al., 2008). According to the time of diagnosis, ischemic perinatal stroke is classified into neonatal ischemic stroke and presumed perinatal ischemic stroke if it is diagnosed after the neonatal period (Raju et al., 2007). Population-based research has estimated the birth prevalence for neonatal AIS to be 1:3,000, presumed AIS to be 1:7,900, and presumed PVI to be 1:6,000 (Dunbar et al., 2020).

Perinatal stroke causes a number of different residual symptoms in later life, which require treatment and rehabilitation (Elgendy et al., 2022). A large prospective study on pediatric AIS has shown that neurological deficits develop in 60% after neonatal AIS, with 39% emerging only at a later follow-up (deVeber et al., 2017). Epilepsy develops in one third of patients with ischemic perinatal stroke (Rattani et al., 2019; Elgendy et al., 2022), and the risk of epilepsy remains elevated for 20 years after stroke (Sundelin et al., 2021).

Children with stroke perform worse in all neuropsychological measures compared to normative expectations, with a younger age at the time of stroke being associated with poorer intellectual functioning (Jacomb et al., 2018). Poorer language skills, social skills, and adaptive behavior have been found in subgroups of perinatal stroke survivors with lower cognitive functions (Anderson et al., 2020). Follow-up at 8 years after stroke showed worse outcomes in attention and executive functioning in children with ischemic stroke compared to children with hemorrhagic stroke, and lower abilities were also seen in most neurocognitive domains (Champigny et al., 2023). Several studies have explored the performance of patients with perinatal stroke in relation to normative means and found lower executive functions and attention performance (Bosenbark et al., 2017; Li et al., 2022). Processing speed, memory, letter fluency, and visual-motor skills have been shown to be worse in children with neonatal and childhood AIS (Abgottspon et al., 2022).

Epilepsy can cause alterations in cognitive abilities. Cognitive and executive functions and attention deficit have been described in different epilepsy syndromes (Cheng et al., 2017; Cainelli et al., 2021; Domańska et al., 2023). Moreover, neuropsychological deficit may already be present at or near the onset of epilepsy (Fastenau et al., 2009; Domańska et al., 2023).

Significantly fewer data have been published on the effect of epilepsy on cognition and particularly its specific sub-functions in patients with perinatal stroke. A recent study of perinatal ischemic stroke, in which active epilepsy and previously diagnosed intellectual disability were excluded, showed that patients with epilepsy had significantly impaired non-verbal intelligence compared with controls (Gschaidmeier et al., 2021). In presumed perinatal stroke, 38% of the patients had epilepsy, and 53% had cognitive impairments associated with epilepsy at the last follow-up (Fitzgerald et al., 2007).

The aim of the present study was to evaluate general and specific cognitive functions in children with epilepsy and children without epilepsy after perinatal ischemic stroke. We hypothesized that children with post-stroke epilepsy have lower general cognitive ability compared to children without epilepsy, while their profile of specific cognitive functions may be variable.

## Materials and methods

This is an observational regional population-based cohort study of patients with ischemic perinatal stroke. The study is part of comprehensive research into the outcome of children with perinatal stroke (Laugesaar et al., 2007, 2018; Ilves et al., 2016, 2022a,b; Lõo et al., 2018; Vaher et al., 2023).

### Participants

Patients were identified from the Estonian Pediatric Stroke Database. The data in this database were collected retrospectively within an epidemiological study from 1994 to 2003 and prospectively from 2004 (Laugesaar et al., 2007, 2018; Vaher et al., 2023). All children with pediatric stroke admitted to the Children's Clinic of Tartu University Hospital are included in the Pediatric Stroke Database. The Children's Clinic of Tartu University Hospital is one of two third-level child neurology centers in Estonia and serves the southern and eastern parts of the country. Patients with perinatal stroke were invited to participate in the study.

The participants of the final study group fulfilled all inclusion criteria: (1) magnetic resonance imaging (MRI)–confirmed diagnosis of unilateral perinatal stroke: neonatal AIS, presumed perinatal AIS, or presumed perinatal PVI; (2) birth ≥36 weeks of gestational age; (3) follow-up 3T MRI scan after neonatal age; (4) cognitive outcome assessment at the age ≥7 years; and (5) electroencephalography (EEG) investigation after neonatal age. The exclusion criteria were as reported earlier: (1) structural disease other than stroke affecting the central nervous system (hypoxic-ischemic encephalopathy, central nervous system's infectious disease, tumor, cortical malformation, or congenital anomaly), (2) specific disease-causing gene variant or copy number variant suggested to be pathogenic for epilepsy or developmental delay, and (3) absence of an MRI confirming stroke (Vaher et al., 2023).

### Clinical data

Clinical information about the pregnancy, the delivery, and the neonatal period was collected from medical records. Pregnancy and birth history and symptoms during the neonatal period, as well as the time of the first epileptic seizure and the time of epilepsy diagnosis, seizure semiology and seizure frequency, and the presence of status epilepticus or electrical status epilepticus in sleep, were recorded as in earlier studies (Ilves et al., 2016, 2022a; Laugesaar et al., 2018; Vaher et al., 2023).

### Neuroimaging

MRI for the outcome study was performed without anesthesia in the chronic stage of perinatal stroke at the age of 6–18 years (*n* = 48). In children who were not able to complete this investigation, an earlier MRI (*n* = 3) performed for clinical purposes at the age older than 1 month was used. A 3T Philips Achieva MRI scanner with an 8-channel SENSE head coil (Philips Medical Systems, Best, The Netherlands) was used.

Based on the time of diagnosis, we classified ischemic perinatal stroke into neonatal ischemic stroke and presumed perinatal ischemic stroke (Raju et al., 2007). Neonatal ischemic stroke is diagnosed after birth, before or on the 28th postnatal day. Presumed perinatal ischemic stroke is diagnosed in infants >28 days of age after the normal neonatal period. In this case, neuroimaging displays signs of chronic infarction, and an ischemic event is presumed to have occurred between the 20th week of fetal life and the 28th postnatal day (Raju et al., 2007; Ilves et al., 2016). According to the anatomical vascular syndrome, ischemic stroke was classified as proximal M1 middle cerebral artery (MCA) infarction, distal M1 MCA infarction, anterior trunk of the distal MCA infarction, posterior trunk of the distal MCA infarction, lenticulostriate infarction, and PVI (Kirton et al., 2008). All radiological images (cerebral ultrasonography, computer tomography, and MRI) of the patients in the Estonian Pediatric Stroke Database, which are stored in the population-based Estonian Picture Archive, were reviewed independently by a radiology resident (No. I) and two neuroradiologists (PI and DL), who were all blinded to the clinical outcome of the patients. The diagnosis of perinatal stroke and the type of vascular genesis were confirmed by consensus agreement.

### Epilepsy and EEG

Post-neonatal EEG was performed in all patients. Standard EEG in the post-neonatal period includes the awake and sleep (daytime nap) periods. An international full 10–20 system was adopted for electrode placement (Kuratani et al., 2016). Epilepsy was diagnosed according to the operational clinical definition of epilepsy proposed by the International League Against Epilepsy (Fisher et al., 2014). One of the following conditions had to be met: (1) at least two unprovoked seizures occurring >24 h apart or (2) one unprovoked seizure with high recurrence risk. All epilepsy diagnoses were reviewed and confirmed by a child neurologist and an EEG specialist (UV). Patients with only interictal epileptiform discharges (IEDs) in the awake or/and sleep state but without a history of clinical seizures were not diagnosed as having epilepsy. Based on the occurrence of epileptic seizures and EEG findings, the patients were divided into three groups: (1) the group with epilepsy—patients with a confirmed epilepsy diagnosis, (b) the group with IEDs—patients with epileptiform activity on EEG but without clinical seizures, and (c) the group without epilepsy—patients without epilepsy and without IEDs. The seizure burden was described using a modified version of the Engel classification: class 0—seizure-free and off anti-seizure medication for >6 months, class 1—seizure-free for at least 6 months while on medication or seizure-free and off medication for < 6 months; class 2—on medication with < 1 seizure a month, class 3–1–4 seizures a month, class 4–5–30 seizures a month, and class 5—>30 seizures a month (Golomb et al., 2007). The electrical status epilepticus in sleep was diagnosed as an EEG pattern of sleep-induced spikes and waves with a frequency of 1.5–3.5 Hz occupying at least 85% of slow sleep (Tassinari and Rubboli, 2006).

### Neurocognitive assessment

The Kaufman Assessment Battery for Children, Second Edition (KABC-II) was employed to assess cognitive outcomes (Kaufman et al., 2005). Three scores of general cognitive ability, the Fluid Crystallized Index, the Mental Processing Index, and the Non-verbal Index, were calculated. Additionally, five subscales, that is, sequential processing, simultaneous processing, learning, planning, and knowledge, were used to evaluate specific cognitive functions and calculate specific index scores for a better understanding of the cognitive profiles.

The sequential processing subscale contains subtests that measure sequential information processing and short-term memory. The simultaneous processing subscale consists of subtests that require simultaneous processing of visual information. Subtests in the learning subscale cover cognitive abilities involved in storing and retrieving newly learned information. The planning subscale contains subtests that measure executive functions, problem-solving, and reasoning abilities. The knowledge subscale relates to verbal abilities and acquired knowledge. Age-appropriate standard scores (*M* = 100, *SD* = ±15) were used for general cognitive ability and specific index scores.

The cognitive assessments were conducted at the Children's Clinic of Tartu University Hospital by a trained clinical psychologist (MM) during a single visit.

### General neurodevelopmental outcome

Neurodevelopmental outcomes were assessed using the Pediatric Stroke Outcome Measure (Kitchen et al., 2012). It contains five subscales: right sensorimotor, left sensorimotor, language production, language comprehension, and cognitive/behavioral performance. The sensorimotor subscales were used to estimate the neurological sequelae of stroke. The outcome was evaluated by one of the two child neurologists (UV, RL).

### Statistics

The statistical package SAS version 9.4 (SAS Institute, Cary, NC) and RStudio were used for statistical analysis. The Shapiro–Wilk test was used for assessing normality. Continuous data were summarized as means with the 95% confidence interval (CI) or medians with the interquartile range (IQR) and categorical data as absolute counts and percentages. The groups were compared using the Kruskal–Wallis test for continuous variables when data were not normally distributed or multinomial logistic regression for dichotomous variables. If significant, *post-hoc* pairwise comparisons were analyzed using the Mann–Whitney *U*-test, χ^2^-test, or Fisher's exact test as appropriate. The odds ratio (OR) with the 95% CI was estimated as the measure of association. The specific cognitive function scores for children with epilepsy and without epilepsy were analyzed using the analysis of variance Student's *t* method or the non-parametric alternative Mann–Whitney *U*-test as appropriate. Multiple testing in each family of tests (eight cognitive function scores) was corrected using the false discovery rate linear step-up procedure (Benjamini and Hochberg, 1995). The Benjamini–Hochberg critical values were calculated as (*i*/*m*)*Q*, where *i* is the rank in an ascending list of *p*-values, *m* is the total number of tests, and *Q* is a false discovery rate of 0.05. Similarly, after significant global tests, *post-hoc* tests were conducted using the Benjamini–Hochberg method. All shown raw *p*-values were two-tailed.

### Ethics

The study was approved by the Research Ethics Committee of the University of Tartu. Written informed consent was provided by all individual participants older than 7 years, as well as by their parents.

## Results

### Population

**Figure 1:**
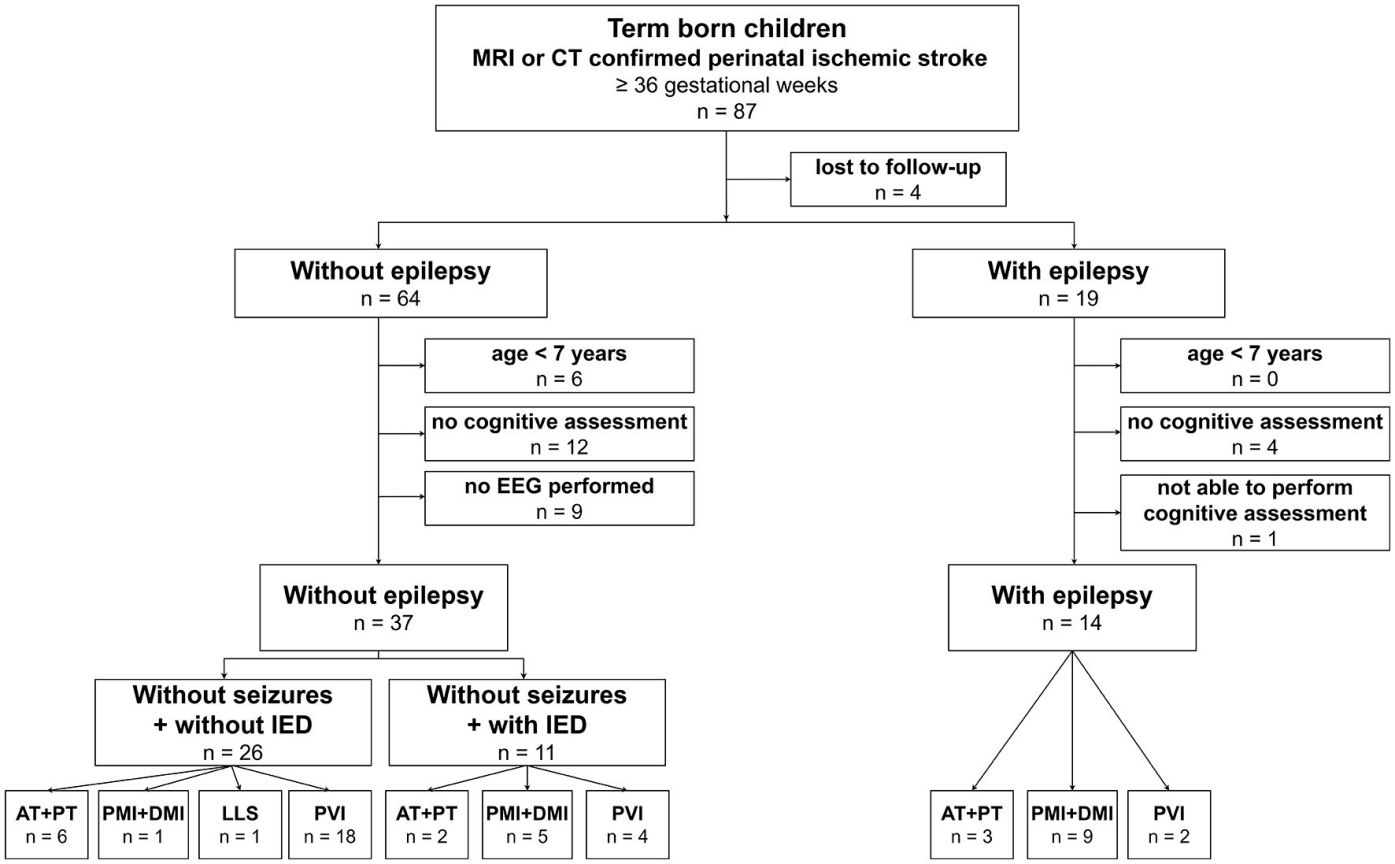
Patient selection. EEG, electroencephalography; IED, interictal epileptiform discharge; PVI, periventricular venous infarction; AT, anterior trunk of the distal middle cerebral artery (MCA) infarction; PT, posterior trunk of the distal MCA infarction; LLS, lenticulostriate arteries infarction; PMI, proximal M1 MCA infarction; DMI, distal M1 MCA infarction.

Eighty-seven patients from the Estonian Pediatric Stroke Database met the criteria for perinatal ischemic stroke and had been born at the age of ≥36 gestational weeks. The detailed flow chart of the study population is presented in Figure 1. The patient who was unable to perform cognitive assessment had severe intellectual disability. She had been diagnosed with PVI and had severe epilepsy with electrical status epilepticus in sleep and recurring status epilepticus.

The final study group consisted of 51 patients: 14 (27.5%) patients with epilepsy and 37 (72.5%) patients without epilepsy. In patients without epilepsy, EEG investigations revealed IEDs in 11 of the 37 (29.7%) patients. The history of these patients did not raise any suspicion of seizures, and an epilepsy diagnosis was not relevant (Figure 1, Table 1).

**Table 1:** Demographics and clinical characteristics.

	**Without epilepsy (*n* = 26)**	**With IEDs (*n* = 11)**	**Epilepsy (*n* = 14)**	***p*-value**	**OR [95% CI]**
Male gender, *n* (%)	13 (50.0)	5 (45.5)	5 (35.7)	0.69	
Gestational weeks at birth, median [IQR]	40 (38–40)	40 (38–40)	40 (39–40)	0.80	
Apgar score at 1 min, median [IQR]	8 (6–9)	8 (7–9)	8 (8–8)	0.93	
	NA = 2		NA = 1		
Apgar score at 5 min, median [IQR]	9 (8–9)	9 (8–9)	8 (8–9)	0.87	
	NA = 2		NA = 1		
Emergency cesarean section, *n* (%)	6/25 (24.0)	2 (18.2)	4 (28.6)	0.91	
	NA = 1				
Neonatal seizures, *n* (%)	3 (11.5)	3 (27.3)	4 (28.6)	0.30	
Side of stroke left, *n* (%)	16 (61.5)	3 (27.3)	10 (71.4)	0.068	
Small for gestational age < 3 percentiles, *n* (%)	3/25 (12.0)	0 (0)	1 (7.1)	0.80	
	NA = 1				
Vascular syndromes, *n* (%)					
Anterior or posterior trunk of MCA infarction	6 (23.1)	2 (18.2)	3 (21.4)	>0.99	
Lenticulostriate arteries infarction	1 (3.8)	0 (0)	0 (0)	>0.99	
Proximal or distal MCA infarction	1 (3.8)	5 (45.5)	9 (64.3)	< 0.0001	< 0.0001^a^, 45 [4.1, 2047]
					0.43^b^
					0.0054^c^, 21 [1.7, 1023]
Periventricular venous infarction	18 (69.2)	4 (36.4)	2 (14.3)	0.0029	0.0009^a^, 14 (2.4-75)
					0.35^b^
					0.080^c^
PSOM general score, median [IQR]	1.5 (1.0–2.5)	3.0 (1.0–4.0)	2.5 (2.5–3.5)	0.083	
PSOM motor score, median [IQR]	1.0 (0.5–1.0)	1.0 (1.0–2.0)	2.0 (0.5–2.0)	0.047	0.049^a^
					0.91^b^
					0.038^c^
Age at KABC-II testing, median [IQR]	9.1 (7.8–10.9)	8.9 (7.2–9.8)	11.1 (7.8–13.2)	0.19	

IEDs, interictal epileptiform discharges; IQR, interquartile range; NA, not available; OR, odds ratio; CI, confidence interval; MCA, middle cerebral artery; PSOM, Pediatric Stroke Outcome Measure; KABC-II, Kaufman Assessment Battery for Children, Second Edition.

Pairwise comparisons were conducted and marked with a superscript.

^a^Epilepsy vs. without epilepsy.

^b^Epilepsy vs. IEDs.

^c^Without epilepsy vs. IEDs.

There were no differences in gestational age, Apgar scores, emergency cesarean section, neonatal seizures, side of stroke, or general Pediatric Stroke Outcome Measure score between the group with epilepsy, the group with IEDs only, and the group without epilepsy (Table 1). There was no difference in the median age at KABC-II testing between the groups.

Children with proximal MCA infarction or distal MCA infarction were more often represented in the group with epilepsy [OR: 45, 95% CI [4.1, 2,047], *p* = 0.0001] and the group with IEDs compared to children with other vascular syndromes [OR: 21, 95% CI [1.7, 1,023], *p* = 0.0054]. Children with PVI were more often represented in the group without epilepsy [OR: 14, 95% CI [2.4, 75], *p* = 0.0009] compared to children with other vascular syndromes (Table 1).

### Clinical features of epilepsy

The median age at the time of the first seizure was 6.2 years (IQR 2.8–7.8; Table 2). Status epilepticus or electrical status epilepticus in sleep occurred in four (28.6%) patients, and eight (57.1%) patients had frequent seizures and needed polytherapy. The median time from the first seizure in 11 of 14 (78.6%) patients who underwent cognitive assessment after the first seizure was 8.2 years (IQR 5.0–9.7). In three patients, a cognitive assessment was performed before the onset of epilepsy at 1.1, 6.7, and 11.8 years before the first seizure, respectively.

**Table 2:** Clinical features of epilepsy.

	**Epilepsy (*n* = 14)**
Age at first seizure, years, median [IQR]	6.2 (2.8–7.8), min 0.3, max 19.6
Age at follow-up for epilepsy, years, median [IQR]	19.3 (14.0–22)
Status epilepticus or electrical status epilepticus in sleep, *n* (%)	4 (28.6%)
Polytherapy, *n* (%)	8 (57.1)
Frequent seizures (Engel class ≥3), *n* (%)	8 (57.1)
Duration of epilepsy at the time of KABC-II testing, years, median [IQR]^*^	8.2 (5.0–9.7)

IQR, interquartile range; KABC-II, Kaufman Assessment Battery for Children, Second Edition.

^*^In 11 of 14 patients whose testing was performed after the first seizure.

### General ability scores for children with epilepsy and children without epilepsy

**Figure 2:**
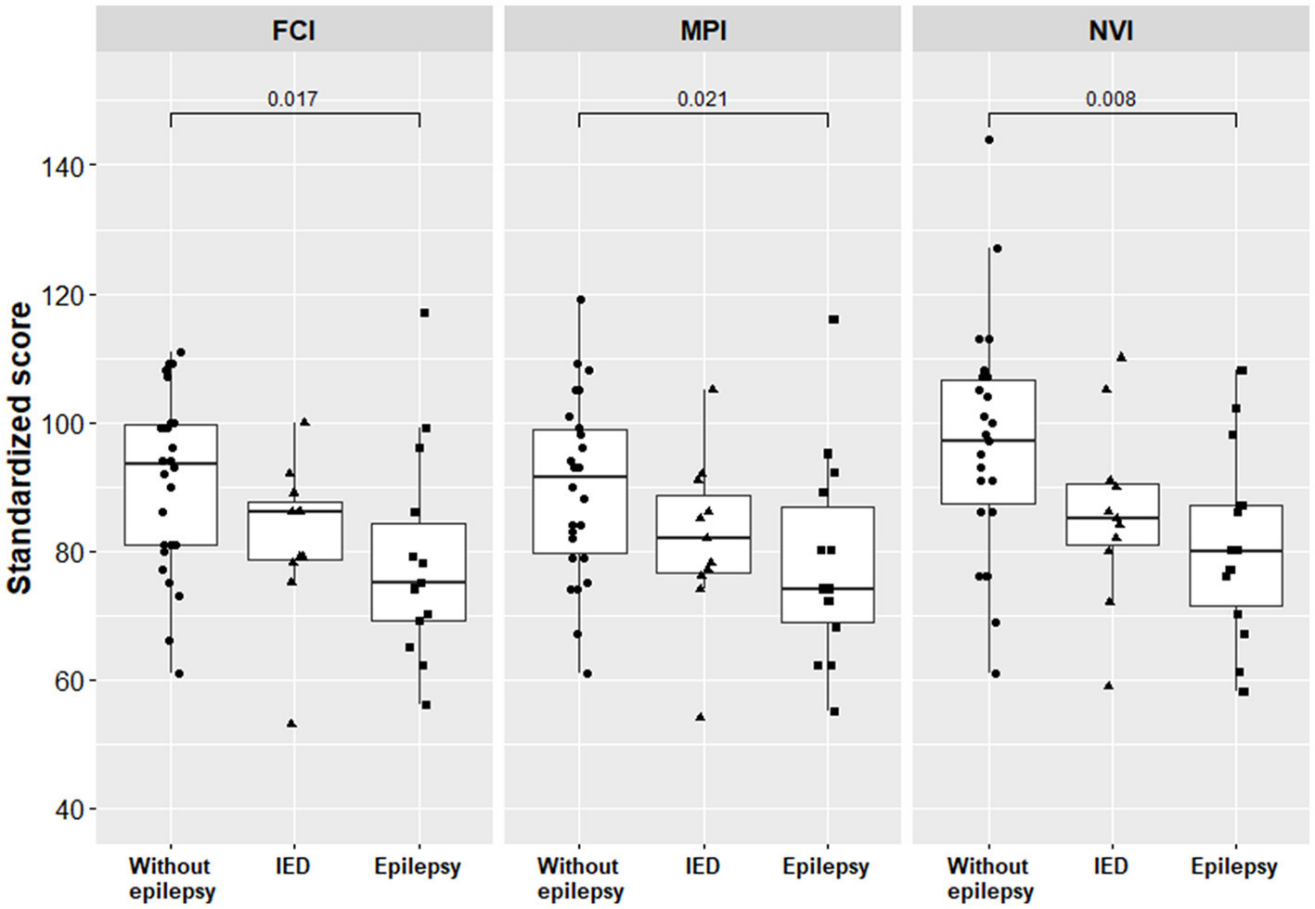
General ability scores for children with epilepsy and for children without epilepsy. Only the *p-*values below the significance threshold of the adjusted false discovery rate are significant and presented in the figure. FCI, Fluid Crystallized Index; MPI, Mental Processing Index; NVI, Nonverbal Index; IED, interictal epileptiform discharge.

All general ability scores (the Fluid Crystallized Index, the Mental Processing Index, the Non-verbal Index) were significantly lower for children with epilepsy compared to children without epilepsy but did not differ between children with epilepsy and children with IEDs only (Table 3, Figure 2).

**Table 3:** Scores of general ability and specific cognitive functions for children with epilepsy and for children without epilepsy.

	**Without epilepsy**	**With IEDs**	**Epilepsy**	**Epilepsy vs. without epilepsy**	**Epilepsy vs. IEDs**	**Without epilepsy vs. IEDs**
	***n* = 26**	***n* = 11**	***n* = 14**	***p*-value**	***p*-value**	***p*-value**
FCI	90.8 [85.2, 96.4]	82.1 [74, 90.2]	78.6 [69.3, 88]	0.017^*^	0.56	0.077
MPI	89.7 [84.1, 95.4]	81.8 [73.2, 90.5]	78.1 [68.9, 87.2]	0.021^*^	0.53	0.12
NVI	96.8 [89.6, 103.9]	85.8 [76.4, 95.2]	81.2 [72.7, 89.8]	0.008^*^	0.44	0.077
SEQ	92.5 [85.4, 99.6]	86.8 [79.2, 94.4]	82.7 [74.3, 91.2]	0.084	0.45	0.33
SIM	96.9 [90, 103.9]	86.2 [73.2, 99.2]	78.5 [69.8, 87.2]	0.0018^*^	0.27	0.10
LEARN	86 (81, 97)	92 (78, 94)	79.5 (75, 92)	0.18	0.30	0.92
PLAN	96.2 [88.7, 103.6]	85.2 [75.2, 95.1]	82.5 [73, 92]	0.026^*^	0.68	0.090
KNOW	92 (84, 108)	89 (87, 92)	80.5 (75, 87)	0.023^*^	0.024	0.24

The data are presented as score points: mean with the 95% confidence interval [e.g., X [Y, Z]] or median with the 25 and 75th percentile [e.g., X (Y, Z)] as statistically appropriate.

FCI, Fluid Crystallized Index; MPI, Mental Processing Index; NVI, Nonverbal Index; SEQ, Sequential Processing; SIM, Simultaneous processing; LEARN, Learning; PLAN, Planning; KNOW, Knowledge; IEDs, interictal epileptiform discharges.

^*^Only the p-values below the significance threshold of an adjusted false discovery rate of 0.038 for epilepsy vs. without epilepsy, 0.0063 for epilepsy vs. IEDs, and 0.0063 for without epilepsy vs. IEDs are significant.

For children with IEDs only, the mean Fluid Crystallized Index score was 9.6% lower compared to children without epilepsy, 82.1 [95% CI [74.5, 90.2]] and 90.8 [95% CI [85.2, 96.4]], *p* = 0.077, respectively; however, this difference did not reach statistical significance at an α level of 0.05. Furthermore, for children with IEDs only, the Non-verbal Index score was 11.4% lower compared to children without epilepsy, but this difference was not statistically different either, 85.8 [95% CI [76.4, 95.2]] and 96.8 [95% CI [89.6, 103.9]], *p* = 0.077, respectively.

### Scores of specific cognitive functions for children with epilepsy and children without epilepsy

**Figure 3:**
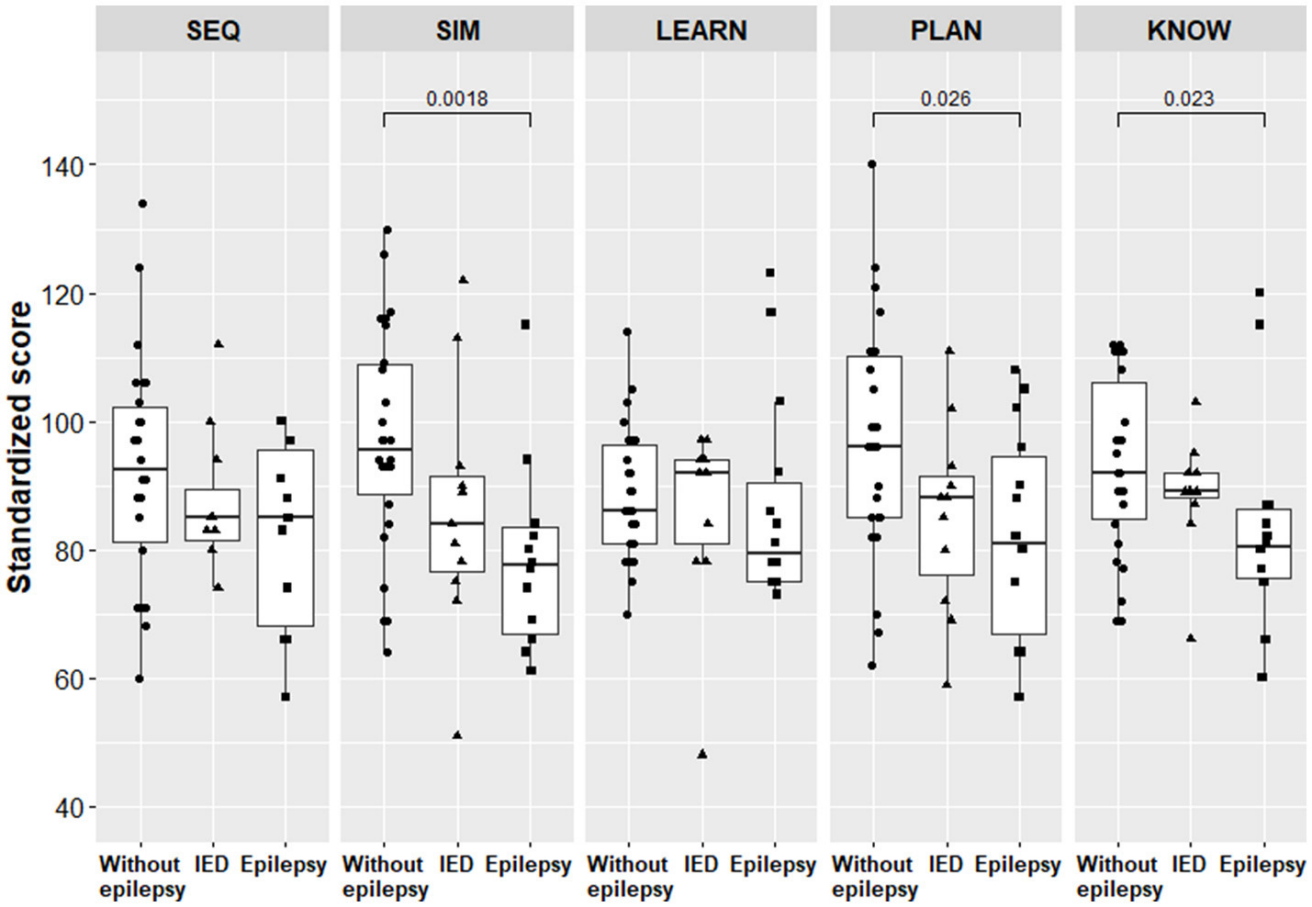
Scores of specific cognitive functions for children with epilepsy and for children without epilepsy. Only the *p-*values below the significance threshold of the adjusted false discovery rate are significant and presented in the figure. SEQ, Sequential Processing; SIM, Simultaneous processing; LEARN, Learning; PLAN, Planning; KNOW, Knowledge; IED, interictal epileptiform discharge.

Three subscale scores, that is, simultaneous processing, planning, and verbal knowledge, were significantly lower for children with epilepsy compared to children without epilepsy (Table 3, Figure 3). The scores of specific cognitive functions did not differ between children with epilepsy and children with IEDs only.

The planning scores were 11.4% lower for children with IEDs only compared to children without epilepsy, with means of 85.2 [95% CI [75.2, 95.1]] and 96.2 [95% CI [88.7, 103.6]], *p* = 0.090, respectively.

### Scores of general ability and specific cognitive functions for children with epilepsy in relation to vascular injury and clinical features of epilepsy

The scores of general ability and specific cognitive functions for children with epilepsy did not differ between children with left-side stroke and those with right-side stroke, *p* ≥ 0.065 (Table 4).

**Table 4:** Differences in scores of general ability and specific cognitive functions for children with epilepsy in relation to vascular injury and clinical features of epilepsy.

	**Anterior or posterior trunk of MCA infarction (*n* = 3) vs. proximal or distal MCA infarction (*n* = 9)**	**Anterior or posterior trunk of MCA infarction (*n* = 3) vs. periventricular venous infarction (*n* = 2)**	**Proximal or distal MCA infarction (*n* = 9) vs. periventricular venous infarction (*n* = 2)**	**Left-side stroke (*n* = 10) vs. right-side stroke (*n* = 4)**	**With SE or ESES (*n* = 4) vs. without (*n* = 10)**	**Polytherapy (*n* = 6) vs. monotherapy (*n* = 8)**	**Engel class ≥3 (*n* = 8) vs. Engel class < 3 (*n* = 6)**
	***p*-value**	***p-*value**	***p-*value**	***p-*value**	***p*-value**	***p*-value**	***p*-value**
FCI	0.16	>0.99	0.72	0.62	0.72	0.37	0.093
MPI	0.14	>0.99	0.81	0.57	0.72	0.40	0.091
NVI	0.041	>0.99	0.41	0.26	>0.99	0.30	0.0199
SEQ	0.31	>0.99	0.81	0.28	>0.99	0.24	0.33
SIM	0.23	0.77	0.12	0.065	>0.99	0.70	0.14
LEARN	>0.99	>0.99	0.72	0.28	0.77	0.74	0.43
PLAN	0.016	>0.99	0.72	0.48	0.72	0.44	0.016
KNOW	0.23	>0.99	0.91	0.89	0.36	0.20	0.36

Pairwise comparison was evaluated by the Wilcoxon–Mann–Whitney test. After correction for multiple testing, the cutoff p-value for the significance of a single comparison for the set of the variables was 0.0063.

FCI, Fluid Crystallized Index; MPI, Mental Processing Index; NVI, Nonverbal Index; SEQ, Sequential Processing; SIM, Simultaneous processing; LEARN, Learning; PLAN, Planning; KNOW, Knowledge; MCA, middle cerebral artery; SE, status epilepticus; ESES, electrical status epilepticus in sleep.

For children with epilepsy and proximal or distal MCA infarction, the Non-verbal Index scores were lower compared to children with anterior trunk or posterior trunk of MCA infarction, *p* = 0.041. These children had also lower planning scores compared to children with anterior trunk or posterior trunk of MCA infarction, *p* = 0.016. After correction for multiple testing, the raw *p*-values were above the new cutoff *p*-value for the significance of a single comparison for the set of variables. There were no differences in the scores of general ability and specific cognitive functions between the groups of PVI and anterior trunk or posterior trunk of MCA infarction and the groups of PVI and proximal or distal MCA infarction.

The Non-verbal Index scores (*p* = 0.0199) and the planning scores (*p* = 0.016) were lower for children who had had frequent seizures (Engel class ≥3) compared to children with rare seizures. After correction for multiple testing, the raw *p*-values were above the new cutoff *p*-value.

There were no differences in the scores of general ability and specific cognitive functions between children who had had status epilepticus or electrical status epilepticus in sleep and children who had not had these complications or between children who received monotherapy and children who received polytherapy.

## Discussion

This study provides a comprehensive assessment of the general ability and specific cognitive functions of children with perinatal ischemic stroke in relation to post-stroke epilepsy. Our data suggest that although the general cognitive ability of children with stroke and epilepsy was lower compared to those who did not develop epilepsy, the scores of specific cognitive functions indicated deficiency only in verbal abilities and executive functions.

According to our study, children with neonatal or presumed perinatal ischemic stroke and post-stroke epilepsy performed worse in all general ability scores compared to children without epilepsy. However, even when data about pediatric stroke and cognition are available, the cognitive functions assessed and the methods used have a large variability, which complicates comparing results. Some studies have compared neonatal or perinatal stroke and childhood stroke and have found lower cognitive skills to be associated with neonatal stroke or younger age at stroke; other studies have assessed children with perinatal and childhood stroke combined but have not considered epilepsy (Jacomb et al., 2018; Anderson et al., 2020; Champigny et al., 2023). A few studies have only focused on neonatal or perinatal stroke, in which case a comparison has often revealed worse performance in cognition compared to normative means (Vojcek et al., 2021; Li et al., 2022). Our data are consistent with the study by Fitzgerald et al., which showed that in perinatal stroke, cognitive impairment was associated with epilepsy at the last follow-up (Fitzgerald et al., 2007). Another study of perinatal ischemic stroke found that patients with epilepsy had only impaired non-verbal intelligence compared to controls, while patients with epilepsy and those without epilepsy did not differ in this respect (Gschaidmeier et al., 2021).

Besides general ability scores, evaluating specific cognitive functions is also important to identify the main field of deficit. We found that the scores for simultaneous processing, planning, and verbal knowledge were significantly lower in children with epilepsy compared to children without epilepsy. Thus, these children performed worse on tasks that require the simultaneous processing of visual information, which measures executive functions, problem-solving, and reasoning abilities and which are related to verbal abilities and acquired knowledge. At the same time, the scores of sequential processing and learning were not different. This shows that children with post-stroke epilepsy still behave equally with children without epilepsy on tasks that measure sequential information processing, short-term memory, and storing and retrieving newly learned information, which allows these strengths to be used in rehabilitation. So far, among the specific cognitive functions affected by epilepsy, the main focus has been on attention and executive functions. Lower results in these domains have been found in different childhood epilepsy syndromes, as well as in patients with pediatric ischemic stroke and children with epilepsy after presumed perinatal AIS (Cheng et al., 2017; Bosenbark et al., 2018; Cainelli et al., 2021; Champigny et al., 2023; Domańska et al., 2023). Our data support the viewpoint about impairment of executive functions, as the planning subscale contains subtests that measure executive functions, which were significantly worse in children with epilepsy compared to children without epilepsy. The cognitive profile of children with post-stroke epilepsy in our cohort is similar to that observed in other childhood-onset epilepsy syndromes, where worse performances have been found across the measures of language and semantic functions and visuomotor functions (Karrasch et al., 2017).

To avoid the possible effect of electrographical discharges on cognitive performance, we separated the children without epilepsy into two groups: children without epileptic seizures and without IEDs and children without epileptic seizures but with IEDs. In our cohort of children with IEDs but without epileptic seizures, we found considerably lower scores on the Fluid Crystallized Index, the Non-verbal Index, and the planning subscale compared to children without IEDs and without seizures. Although the differences were not statistically significant, we suggest that this impairment may be important clinically and should be considered in clinical practice. At the same time, no differences were seen in the general cognitive ability and specific cognitive function scores between patients with epilepsy and patients with IEDs only. Previous studies of childhood epilepsies have shown that diurnal IEDs in ≥10% of the EEG records are related to impaired central information processing speed, short-term verbal memory, and visual-motor integration, and in patients with perinatal stroke, electrical status epilepticus in sleep (determined as spike-and-wave discharges occurring in >75% of sleep) is associated with worse scores in receptive language and externalizing behaviors (Ebus et al., 2012; Mineyko et al., 2017).

In the group of children with epilepsy, we analyzed the differences in cognitive scores according to the vascular syndrome and the side of stroke. The Non-verbal Index scores and planning subscale scores were lower in children with proximal or distal MCA infarction compared to children with anterior or posterior trunk of MCA infarction. After corrections for multiple testing, this difference was no longer statistically significant, but our results were compatible with a study of neonatal AIS, where the main middle cerebral artery stroke was related to overall worse outcomes and epilepsy (Vojcek et al., 2021). There were no differences in the cognitive profile between children with different vascular syndromes of AIS and PVI. However, we have to bear in mind that one child with PVI was unable to perform the tests due to severe intellectual disability and was excluded from the study group. Regarding the side of stroke, we did not find differences between the children with left-side stroke and right-side stroke, which is consistent with previous data (Van Buuren et al., 2013; Gschaidmeier et al., 2021).

Of the clinical indicators, we found lower Non-verbal Index scores and lower planning subscale scores for children with frequent seizures compared to children with rare seizures. It has been shown that in the case of childhood epilepsies, patients with continuing seizures perform worse not only in language and semantic functions but also in visuomotor functions (Karrasch et al., 2017). In our cohort of patients with epilepsy, the presence of the status epilepticus or electrical status epilepticus in sleep or the use of polytherapy did not affect cognitive scores. Although it has been shown that anti-seizure medication, and especially polytherapy, has an effect on neuropsychological domains, epilepsy itself and its etiology are the main contributors to cognitive impairment, as cognitive changes may already be present at the onset of epilepsy (Fastenau et al., 2009; Anderson et al., 2015; Matricardi et al., 2016).

The mechanism behind the different patterns of changes in specific cognitive functions in children with perinatal stroke with epilepsy is not clear. In a study of carefully selected patients with left-side medium to large perinatal AIS but without epilepsy, all language tasks showed completely normal language abilities (Newport et al., 2022). Functional imaging tasks revealed an activation in the frontal and/or temporal regions of the right hemisphere, homotopic for healthy controls (Newport et al., 2022). Laterality indices showed that all patients but one, with the smallest lesion of perinatal stroke, were right-lateralized for sentence processing, demonstrating the ability of the young brain to reorganize language (Newport et al., 2022). The data from another functional imaging study of perinatal stroke patients in which epilepsy patients were included confirm that language activation is reorganized to the right hemisphere in patients with large AIS lesions, but this did not ensure normal language outcomes (Ilves et al., 2022b). A neuropsychological and functional study of children with frontal and temporal lobe epilepsies without significant structural abnormalities discovered reduced connectivity within the left hemisphere, as well as between hemispheres; increased connectivity within the right hemisphere; and higher right hemispheric local efficiency for the epilepsy group compared to the control group (Hüsser et al., 2023). Patients with epilepsy performed worse in cognitive domains compared to patients without epilepsy, which suggests that brain reorganization in response to epilepsy does not allow for optimal cognitive development (Hüsser et al., 2023). These results suggest that, besides anatomical changes, functional network alterations exist that affect patients' outcomes. This probably happens in the case of perinatal stroke with epilepsy. Functional studies in perinatal stroke patients with epilepsy are needed to find out the mechanisms underlying the development of epilepsy.

Our study has important implications for understanding the cognitive performance in children with perinatal stroke and epilepsy. The results of the study indicate that according to the general cognitive ability scores, children with post-stroke epilepsy perform worse compared to children without epilepsy. At the same time, sequential information processing, short-term memory, and storing and retrieving newly learned information remain at the same level as in children without epilepsy. Although we found lower values also in some cognitive domains in patients with IEDs but without seizures compared to patients without epilepsy, studies with larger samples are needed to demonstrate statistically significant changes in this case. In patients with perinatal stroke and particularly in patients with post-stroke epilepsy or with IEDs only, in addition to general cognitive evaluation, cognitive functions covering different domains should be assessed and targeted rehabilitation be established.

We should acknowledge some limitations of our study. First, some study groups, especially the group with IEDs, were too small to reveal statistically significant differences. Second, the duration of epilepsy at the time of KABC-II testing varied. However, we opted for assessment at the age of ≥7 years to evaluate not only general ability but also as many specific cognitive functions as possible. In many cases, epilepsy begins in the 1st years of life, but comprehensive evaluations of cognition at this age are limited. Third, we cannot rule out the possible effect of medication on cognitive performance in children with epilepsy. Still, we showed that there was no difference in cognitive ability scores between children receiving monotherapy and children receiving polytherapy.

Further research should focus on longitudinally evaluating general ability and specific cognitive functions in not only children with post-stroke epilepsy but also in children with IEDs without clinical seizures. More research is also required to determine the interaction of variables other than epilepsy with cognitive performance in children with perinatal stroke.

## Conclusion

Children with perinatal ischemic stroke and post-stroke epilepsy had lower general cognitive ability scores compared to children with stroke but without epilepsy. The profile of the subscales revealed lower scores for simultaneous processing, planning, and knowledge in children with epilepsy compared to those without epilepsy, which indicates deficiencies in verbal abilities and executive functions. Our data highlight the importance of assessment of not only general ability but also special cognitive abilities in patients with perinatal stroke. This would allow targeted rehabilitation to be provided by making use of less impaired cognitive functions.

## Data availability statement

The raw data supporting the conclusions of this article will be made available by the authors, without undue reservation.

## Ethics statement

The studies involving humans were approved by Research Ethics Committee of the University of Tartu. The studies were conducted in accordance with the local legislation and institutional requirements. Written informed consent for participation in this study was provided by the participants' legal guardians/next of kin.

## Author contributions

UV: Conceptualization, Data curation, Investigation, Methodology, Writing – original draft, Writing – review & editing. MM: Conceptualization, Data curation, Investigation, Methodology, Supervision, Writing – review & editing. RL: Data curation, Investigation, Writing – review & editing. NoI: Investigation, Writing – review & editing. NiI: Investigation, Writing – review & editing. DL: Investigation, Writing – review & editing. PK: Formal analysis, Software, Visualization, Writing – review & editing. PI: Conceptualization, Data curation, Funding acquisition, Investigation, Methodology, Project administration, Resources, Supervision, Writing – review & editing.
